# People with diabetes and ambulance staff perceptions of a booklet-based intervention for diabetic hypoglycaemia, “Hypos can strike twice”: a mixed methods process evaluation

**DOI:** 10.1186/s12873-022-00583-y

**Published:** 2022-02-08

**Authors:** Despina Laparidou, Vanessa Botan, Graham R. Law, Elise Rowan, Murray D. Smith, Amanda Brewster, Robert Spaight, Pauline Mountain, Sally Dunmore, June James, Leon Roberts, Kamlesh Khunti, A. Niroshan Siriwardena

**Affiliations:** 1 Community and Health Research Unit and Lincoln Clinical Trials Unit, School of Health and Social Care, University of Lincoln, Brayford Pool, Lincoln, Lincolnshire LN6 7TS UK; 2 Patient and Public Contributor, Lincoln, UK; 3 Clinical Audit and Research Unit, East Midlands Ambulance Service (EMAS) NHS Trust, Nottingham, UK; 4 University Hospitals of Leicester NHS Trust, Leicester, UK; 5 Leicester Diabetes Centre, University of Leicester, Leicester, UK

**Keywords:** Diabetes, Hypoglycaemia, Booklet-based intervention, Process evaluation, Ambulance staff, Perceptions, Experiences

## Abstract

**Background:**

Hypoglycaemia is a potentially serious condition, characterised by lower-than-normal blood glucose levels, common in people with diabetes (PWD). It can be prevented and self-managed if expert support, such as education on lifestyle and treatment, is provided. Our aim was to conduct a process evaluation to investigate how ambulance staff and PWD perceived the “Hypos can strike twice” booklet-based ambulance clinician intervention, including acceptability, understandability, usefulness, positive or negative effects, and facilitators or barriers to implementation.

**Methods:**

We used an explanatory sequential design with a self-administered questionnaire study followed by interviews of people with diabetes and ambulance staff. We followed the Medical Research Council framework for process evaluations of complex interventions to guide data collection and analysis. Following descriptive analysis (PWD and staff surveys), exploratory factor analysis was conducted to identify staff questionnaire subscales and multiple regression models were fitted to identify demographic predictors of overall and subscale scores.

**Results:**

113 ambulance staff members and 46 PWD completed the survey. We conducted interviews with four ambulance staff members and five PWD who had been attended by an ambulance for a hypoglycaemic event. Based on surveys and interviews, there were positive attitudes to the intervention from both ambulance staff and PWD. Although the intervention was not always implemented, most staff members and PWD found the booklet informative, easy to read and to use or explain. PWD who completed the survey reported that receiving the booklet reminded and/or encouraged them to test their blood glucose more often, adjust their diet, and have a discussion/check up with their diabetes consultant. Interviewed PWD felt that the booklet intervention would be more valuable to less experienced patients or those who cannot manage their diabetes well. Overall, participants felt that the intervention could be beneficial, but were uncertain about whether it might help prevent a second hypoglycaemic event and/or reduce the number of repeat ambulance attendances.

**Conclusions:**

The ‘Hypos may strike twice’ intervention, which had demonstrable reductions in repeat attendances, was found to be feasible, acceptable to PWD and staff, prompting reported behaviour change and help-seeking from primary care.

**Trial registration:**

Registered with ClinicalTrials.gov: NCT04243200 on 27 January 2020.

**Supplementary Information:**

The online version contains supplementary material available at 10.1186/s12873-022-00583-y.

## Background

Hypoglycaemia is a potentially serious condition, characterised by lower-than-normal blood glucose levels, common in people with diabetes [1]. Hypoglycaemia has immediate (e.g., palpitations, confusion, loss of consciousness, etc.) but also long-term physical (e.g., cardiovascular, neurological), psychological (e.g., cognitive decline) or social (e.g., affecting work, driving or quality of life) effects on those affected [2] and family members [3] so it needs prompt recognition and intervention.

Severe hypoglycaemia (defined here as hypoglycaemia requiring third party assistance) is often treated by the carers of the person with diabetes (PWD) but in some cases requires Emergency Medical Services (ambulance) attendance [4]. Recurrent episodes may also require ambulance attendance or hospitalisation putting pressure on services and increasing costs [5]. However, hypoglycaemia can be prevented and self-managed if expert support is provided [6]. Education on lifestyle (e.g., diet, exercise) and treatment plays an important role in the prevention of hypoglycaemic episodes and lack of awareness of hypoglycaemia which can complicate it [7].

Patient information leaflets/booklets have been shown to be an efficient means of educating people with diabetes [8, 9]. Kumaran et al. [8] and Sankar et al. [9] identified that leaflets could improve knowledge and confidence, which helped PWD to adhere to treatment and better manage their condition. Conversely, leaflets may be difficult to read or understand for some (e.g., older) PWD [10] or add to time on scene for ambulance staff.

“Hypos can strike twice” (HS2) is part of a complex quality improvement intervention involving ambulance staff providing treatment and advice to people who have had a hypoglycaemic attack to access follow-up care by the general practitioner (GP) or specialist diabetes team, supported by the provision of an educational HS2 booklet, which the PWD can read when they are fully recovered from the cognitive and other effects of the hypoglycaemic episode (See Additional file 1 for a copy of the HS2 booklet). Its main aim is to prevent repeat ambulance calls and attendances for hypoglycaemia. A recent quasi-experimental study conducted in the East Midlands region of the United Kingdom (UK) demonstrated positive effects of the “Hypos can strike twice” (HS2) intervention, including significantly reduced numbers of ambulance attendances for recurrent hypoglycaemic events and improved care processes [11].

We wished to understand how the HS2 intervention was implemented, how it achieved its effects, and the contextual factors external to the intervention itself that could affect implementation, sustainability, and potential for wider roll-out into standard practice [12]. Our aim was to conduct a process evaluation to investigate how ambulance staff and PWD perceived the “Hypos can strike twice”, including acceptability, understandability, usefulness, positive or negative effects, and facilitators or barriers to implementation.

## Methods

### Study design

We used an explanatory sequential design with a self-administered questionnaire study followed by interviews of PWD and ambulance staff.

### Process evaluation framework

We used the Medical Research Council (MRC) framework for process evaluations of complex interventions to guide data collection and analysis [12]. This included understanding: the intervention and its causal assumptions; the implementation process (how delivery was achieved, training, resources, etc.) and what was delivered (fidelity, adaptations, etc.); the mechanisms of impact (participant responses to and interactions with the intervention, mediators, unexpected pathways and consequences); context (contextual factors that shape theories of how the intervention works and that affect the implementation, intervention mechanisms, etc.); and outcomes [12].

### Settings and participants

The intervention was implemented across the East Midlands, UK between December 2018 and November 2019 and data were collected from September 2020 to March 2021. Inclusion criteria for ambulance clinicians were those staff who were actively providing treatment and advice to people who had experienced a hypoglycaemic event during the study period and excluded staff who had not. PWD were included if they were aged 16 years or over, had diabetes and experienced hypoglycaemia needing an ambulance service response during the study period, while excluding those people with diabetes aged under 16 years, who had called an ambulance for reasons other than hypoglycaemia.

Hypoglycaemia for this study was identified as a clinical diagnosis (recorded as a ‘clinical impression’ on the electronic ambulance record) of ‘hypoglycaemia’ or ‘diabetic problem’. Recruitment was initially conducted by a researcher working at East Midlands Ambulance Service (EMAS), who contacted all eligible PWD (using EMAS clinical records data of incidents categorised above) and frontline EMAS staff members.

Potential PWD participants received a study pack (cover letter, participant information sheet, consent form, questionnaires in paper format) by post, whereas potential staff participants received the study pack (including a link to the online questionnaire) by email. The study was also advertised to ambulance staff on EMAS social media pages.

At the end of the questionnaires participants had the opportunity to express their interest in a follow-up interview to discuss their experience with receiving (PWD) or implementing (staff members) the HS2 intervention. A researcher from the University of Lincoln, UK then contacted interested participants directly to arrange and conduct the interviews.

### Questionnaire development and structure

Survey items (for both PWD and staff members) were constructed following the MRC framework domains (implementation process, mechanisms of impact, context, and outcomes) [12]. The staff survey comprised 33 items covering staff satisfaction with the HS2 intervention and with the training they had received (Table S1; see Additional file 2). The PWD survey (Table S2; see Additional file 2) comprised 29 items, covering PWD’ perceptions and experiences of participating in the intervention. For both surveys, we included a balanced number of both negatively and positively framed statements as questions/items. Items were scored on a 5-point Likert scale.

### Statistical analysis

Descriptive statistics were used to summarise survey responses. Box and whisker plots were used to summarise the distribution of responses showing median and interquartile range (IQR) for each item for the PWD survey and for each questionnaire subscale for the staff survey. Negatively framed items in the staff (Table S3; see Additional file 2) and PWD survey (Table S4; see Additional file 2) were reverse scored, so that higher scores indicated more positive attitudes.

Exploratory factor analysis using varimax rotation was conducted on the staff survey after checking if factor analysis was applicable (KMO > 0.7). Cronbach’s alpha was calculated for internal consistency of identified factors (subscales) and for surveys as a whole. Multiple regression models were fitted to explore independent associations between staff demographic characteristics and perceptions. *P* values lower than 0.05 were considered statistically significant. The main outcomes were represented by each subscale (or factor) and by the questionnaire’s overall score. Data were analysed using the statistical software Stata 15.1.

### Qualitative data collection and analysis

The purpose of the qualitative interviews was to provide a richer understanding of PWD and staff perceptions of the ‘Hypos can strike twice’ intervention, using the complex intervention process evaluation framework to construct the interview schedule and guide data analysis and synthesis.

Semi-structured interviews were conducted online (via Microsoft Teams), due to restrictions imposed by the Covid-19 pandemic. We also included in this qualitative data analysis the answers to the free-text questions from the PWD and survey questionnaires, where participants were asked “If there is anything else you would like to tell us about your experience with the booklet, please do so here”. The audio recorded data were transcribed verbatim and were entered into NVivo 12 qualitative data analysis software to facilitate analysis. Two researchers (DL, NS) read and re-read transcripts, which were then coded (by DL) and discussed with other members of the research team to then, through an iterative process, organise codes, develop descriptive and identify analytic themes.

### Integration of quantitative and qualitative results

We integrated quantitative and qualitative data following the MRC Framework for process evaluation domains [12]. Our aim was to triangulate findings, by comparing and contrasting results, as well as finding similarities between the two different sets of data. We also aimed to ‘explain’ the quantitative findings by looking in more depth into the participants’ perceptions of and experiences with the intervention during the interviews. To present the integrated results we used both a narrative synthesis and a joint display [13, 14].

## Results

### Survey responders

In total, 113 ambulance staff members completed the survey of whom 63 completed it partially; 46 PWD completed the survey. Participant demographics are shown in **Tables S1** (staff) **and S2** (patients) in supplementary materials and results (see Additional file 2).

### Staff survey findings

According to staff responses, the booklet was given out to PWD in about half the episodes of hypoglycaemia call-outs they attended. Moreover, about half of the staff considered it was easy to implement the intervention and expressed confidence implementing it (Table 1). Staff responses to the remaining questionnaire items indicated that the intervention was generally well received. A detailed account of each item and staff answers can be found in **Table S3** in supplementary materials and results (see Additional file 2). The reliability coefficient (Cronbach’s α) was 0.89 indicating good consistency amongst the items.

**Table 1:** Number and percentage of staff reporting on the frequency and ease of implementing the booklet

	Never	Rarely	Sometimes	Often	Always	Median [Interquartile range]
1. How often did you give out the leaflet to patients with hypos?	12 (15.0%)	13 (16.3%)	14 (17.5%)	18 (22.50%)	23 (28.75%)	**4 [2,5]**
2. How often did you complete all the sections of the leaflet?	15 (18.75%)	5 (6.25%)	8 (10.00%)	15 (18.75%)	37 (46.25%)	**4 [2.5, 5]**
	**Very Difficult**	**Slightly Difficult**	**Neutral**	**Easy**	**Very Easy**	
3. How easy/difficult was it to implement the HS2?	3 (3.90%)	7 (9.10%)	29 (37.66%)	26 (33.77%)	12 (15.58%)	**3 [3,4]**
	**Not at all**	**Slightly**	**Moderately**	**Very**	**Extremely**	
4. How confident were you implementing the intervention?	9 (11.39%)	8 (10.13%)	18 (22.78%)	35 (44.30%)	9 (11.39%)	**4 [3,4]**

**Fig. 1:**
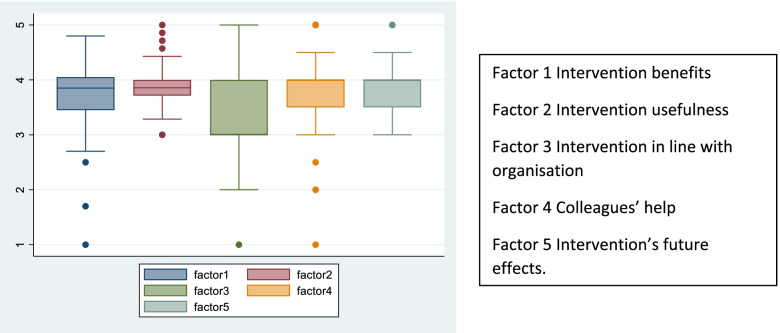
How the intervention was perceived by staff

A subsequent factor analysis identified five main subscales (or factors) with eigenvalues higher than 1. All subscales had good (> 0.7) or acceptable (> 0.6) internal consistency, presented in Table 2. The overall scores for each theme indicated positive attitudes towards the intervention and are represented in Fig. 1*.*

**Table 2:** Factors, component items and internal consistency of staff survey

Factor	Items	Cronbach’s alpha
Factor 1: Intervention benefits	Q5, Q8, Q9, Q10, Q13, Q14, Q17, Q28, Q30, Q32	0.93
Factor 2: Intervention usefulness	Q6, Q7, Q11, Q12, Q18, Q25, Q33	0.80
Factor 3: Intervention in line with organisation culture	Q15, Q16	0.93
Factor 4: Colleagues’ help	Q26, Q27	0.86
Factor 5: Intervention’s future effects	Q29, Q31	0.61

Boxplots indicate medians and IQRs for each factor. Higher values indicate more positive attitudes.

The results indicated that gender and qualification were both significant predictors of Intervention’s future effects.

Female ambulance staff and Emergency Medical Technicians were less optimistic about intervention’s future effects compared with male and paramedic staff respectively. Detailed results can be seen in Table 3.

**Table 3:** Predictors of staff survey factors and overall outcome

Predictors	Outcomes (factors)					
	Intervention benefits	Intervention usefulness	Intervention in line with organisation culture	Colleagues’ help	Intervention’s future effects	Overall Satisfaction
	Coef. (95% CI)	Coef. (95% CI)	Coef. (95% CI)	Coef. (95% CI)	Coef. (95% CI)	Coef. (95% CI)
**Gender**	0.45 (− 0.09,0.99)	−0.1 (− 0.44, 0.25)	− 0.01 (− 0.63, 0.62)	0.28 (− 0.24, 0.81)	− 0.32* (− 0.63, − 0.02)	0.14 (− 0.21, 0.49)
**Age**	− 0.15 (− 0.53,0.23)	− 0.1 (− 0.35, 0.15)	− 0.01 (− 0.47, 0.44)	−0.13 (− 0.50, 0.25)	0.002 (− 0.22, 0.22)	−0.10 (− 0.34, 0.14)
**Qualification**	0.01 (− 0.27,0.28)	− 0.01 (− 0.18, 0.16)	−0.01 (− 0.33,0.30)	0.04 (− 0.23, 0.30)	−0.19* (− 0.34, − 0.04)	−0.001 (− 0.18, 0.18)
**Years of experience**	− 0.03 (− 0.27,0.22)	0.07 (− 0.09, 0.23)	0.07 (− 0.23, 0.36)	0.13 (− 0.11,0.38)	0.03 (− 0.11, 0.18)	0.03 (−0.12, 0.19)
**Personal history of Diabetes**	0.01 (−0.51,0.52)	−0.1 (− 0.44, 0.23)	−0.003 (− 0.62,0.62)	0.02 (− 0.5, 0.53)	0.13 (− 0.17, 0.44)	−0.01 (− 0.34, 0.31)
**Participation in other interventions**	− 0.13 (− 0.48,0.20)	−0.03 (− 0.25, 0.19)	−0.03 (− 0.43, 0.38)	−0.2 (− 0.52, 0.15)	0.17 (− 0.03, 0.37)	−0.07 (− 0.28, 0.14)

*p < 0.05

### PWD survey findings

**Fig. 2:**
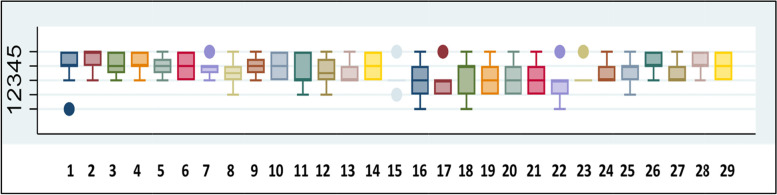
Score distribution represented by medians and IQRs for each questionnaire items of the PWD survey

Almost one third of responders (29.3%) reported receiving the booklet. The PWD survey had good internal consistency (Cronbach’s α =0.97) and indicated that the intervention was generally well received by PWD. The overall score for each item was around or above the median (of 3), showing that the intervention was considered beneficial by PWD (Fig. 2**)***.* The items that received the lowest scores were items 16 to 22, which assessed a change in behaviour such as consulting the GP more often, changing diet, drinking less alcohol etc. Detailed responses to each item can be seen in Table S4 in supplementary materials and results (N.B. only 10 PWD answered these items) (see Additional file 2).

Boxplots indicate medians and IQRs for each item of the PWD survey, higher values indicating more positive attitudes. Detailed description of each item can be found in Table S4, in supplementary materials and results (see Additional file 2).

### Qualitative results

#### Participant characteristics

We conducted interviews with four ambulance staff members and five PWD with diabetes who had been attended by an ambulance for a hypoglycaemic event.

Staff interviewees mostly self-identified as male (3/4), were all of White ethnicity and their ages ranged between 20 and 55 years. Two were paramedics, one an Emergency Medical Technician (EMT), and the fourth an EMT and student paramedic. Staff interviews lasted between 20 and 49 min.

All PWD self-identified as female and white British or Irish. Their ages ranged between 35 and 80 years. Two PWD remembered being offered the ‘Hypos can strike twice’ booklet by the ambulance staff, whereas three were not offered or could not remember being offered the booklet. PWD interviews lasted between 13 and 43 min.

We also included free text responses (FTR) to the staff and PWD survey. Quotes from interviews and survey responses are denoted ambulance staff (AS) or PWD (P).

#### Themes

After initial coding and development of descriptive themes, we developed five analytical themes, following the four key components and functions of the MRC framework (implementation, mechanisms of impact, context, outcomes) [12], and a fifth theme describing the participants’ suggestions for future development of the HS2 booklet intervention.

#### Implementation process

Ambulance staff members reported successfully implementing the intervention as instructed (fidelity) with most eligible PWD:

“After I’ve finished, obviously, all the treatments and the patient’s hypo has been reversed and they’re able to understand what I’m talking about, then I’d be able to give all the further information and further advice using the leaflet, record it and point them to the bits on it that will be pertinent, and obviously fill that out and then leave it with them so they can use it in future, with the idea of preventing further hypos.” AS4

“We always make sure we’ve got some on the vehicle readily available, and if we’re going in to a diabetic, we take it in with us straight away with all the other equipment that we take in. So, we always fill it out; we always hand it out; we always make sure we’ve got them.” AS2

On the few occasions that the intervention was not implemented the main reason given was not attending eligible PWD or the PWD’ condition that had deteriorated rapidly:

“And then he began to show signs and symptoms again, despite his blood glucose remaining at a satisfactory, safe level. [ … .] Then, in the end, we just called it a day, put ‘Hypos strike twice’ to bed, and ended up conveying him.” AS3

A few staff members, however, reported seeing booklets at their station, but not being told that these booklets were part of an intervention nor that they were supposed to be giving them out to PWD experiencing a hypoglycaemic event:

“Leaflets seemed to just ‘appear’ on vehicles and not many knew why or what to do with them etc. I received no training or induction on the leaflets.” ASFTR1

As a result, these staff members had never implemented (or witnessed anyone implementing) the intervention; taking part in the survey, though, had prompted them to start handing booklets out to eligible PWD:

“I cannot comment on most of these questions one way or another as I have not yet used the leaflets as they were designed for. I will commence using them as a result of this survey.” ASFTR2

Ambulance staff also reported not receiving training on the intervention but felt it was not necessary since they found the booklets “self-explanatory”:

“I hadn’t actually been trained in its use, but I don’t think it was really necessary. These leaflets were just on our ambulance, they were self-explanatory, and my crewmate knew about them if I needed any guidance.” AS3

Those who reported receiving training felt it was timely and relevant:

“Yeah, I felt the training was timely and relevant. We had … as I say, it was only a few hours as far as I can remember, it wasn’t a full day, so the timing was fine. It was related to what we needed to do, everything was in place for us to then go ahead and do it.” AS1

Finally, staff were reassured knowing they could rely on colleagues or contact the study team for further guidance:

“ … .it was fine by me and I managed to do the study okay, and if there were any problems, I know we had a contact number and name to contact if we had any queries.” AS1

#### Mechanisms of impact

Ambulance staff interviewees who implemented the intervention reported being positive overall about the intervention and its implementation and felt it was being implemented by other staff as well:

“I think it’s well received, and you do run out of them on the vehicle sometimes, which would suggest that they are used, which is good.” AS3

They also reported that most PWD were happy and found it useful to receive the booklet and advice from ambulance crews:

“Yeah, I’d say about three quarters, three out of four are positive and welcome the advice. A lot of them have fed back that they’ve had a diagnosis from the GP of diabetes, but they’ve not really had anyone sit down and talk to them about the specifics, you know the diet and what they should do in the event of a hypo and things like that. So, they definitely find going over it and having something written down that they can keep useful.” AS4

Some reported mixed views with certain staff not being as receptive, as they felt the booklet meant extra work for them:

“Some people say it’s a waste of time. They say the patient’s not bothered about it. They say it’s extra work for them to do. Other people are more keen and happy to write out more information to cover themselves, to help the patient, for adequate safety netting. Generally, I found most people don’t fill it out from my experience working with them.” AS4

Some also felt PWD were not always receptive to advice or that the booklet was ‘too much to read’:

“Patients frequently had poor willingness to engage in any advice written or verbal given to them post active treatment. Several patients put the leaflet straight into the bin saying it was too much to read.” ASFTR3

This was especially true for newly diagnosed PWD who were “in denial” or PWD who had diabetes for many years and were experienced at handling their condition:

“I think on the odd occasion, you get the stroppy teenager or the in-denial diabetic that’ll say, ‘Oh, yeah, just more paperwork’ or ‘Oh, another leaflet’, that sort of thing. But that’s probably their own hang-ups that they’ve got with being diabetic, especially in younger ages and young adults who are struggling with accepting the fact that they’ve got diabetes, which is very hard for some of them.” AS2

“A number of patients were in fact offended at the idea of the leaflet as they've had the condition for many years.” ASFTR4

Similar views were also expressed by PWD, who felt that the booklet would be more useful to someone with less experience (all interviewed PWD had been diagnosed for many years) or who could not manage their diabetes well:

“If I had been issued the leaflet, I probably wouldn’t have looked at it because I know what I’m doing.” P2

“I don’t remember learning anything new from the leaflet, but it would certainly have been useful to somebody who had been recently diagnosed or was having problems getting adjusted to the condition and any changes in lifestyle they had to make.” P3

Ambulance staff generally felt that there was a need for such an intervention, both for safety-netting, as well as a reminder for the PWD to be cautious to prevent a repeat hypoglycaemic event:

“Like I say, it encourages us to leave safety-netting behind and it highlights to the patient that it can happen again, they should be mindful of the warning signs, that sort of thing … So, in that sense, very beneficial.” AS3

Ambulance staff also felt that the intervention was easy to implement, that it fitted well with their existing role and workload, and that it enhanced or complemented care:

“It doesn’t take long to fill in; it’s very easy; it’s not difficult. So, I don’t see a problem with it.” AS2

“It filled a gap because I wouldn’t have felt right leaving them with no documentation. So, I would’ve done my own write-up for them and left it with them, so that if the same thing happened quickly after or again in the future, any other responding crews will have the documentation right there in front of them outlining how we treated it and how they responded. So, bearing in mind I was going to do some writing anyway, finding the ‘Hypos strike twice’ documents just gave me the framework to write out a story basically, for any other crews coming to the same patient.” AS3

“I’d say it ensured it was more detailed with the safety netting and the ongoing advice on how they can go about preventing further hypos and it just generally prompts you to give more information.” AS4

In addition, staff members also reported finding the booklet easy to read and understand, informative, useful as a record of the care they provided, and an addition to or continuation of their care:

“It’s never a problem and reading them, they’re pretty self-explanatory: there’s nothing in there that’s difficult to read; it’s quite easy to go through; it’s understandable to read; it’s not complicated; it doesn’t speak in high language; it’s for everybody to understand. I don’t think I’ve ever had to explain anything in it, it’s pretty self-explanatory.” AS2

“I feel it is a useful tool to be able to utilise with patients as it allows us to give the advice as well as leaving something for them to read and take away from the visit to encourage them to take more responsibility for themselves” ASFTR5

“I think interventions like this one are really useful because it just adds a material level to what we’re telling them and also lasts longer than our advice. Because, while we’re there, they might not take everything in, because some people, you know, 999 has been called and they’re a bit stressed, they might not be thinking about everything, and they’ve just recovered from a hypo, so they might not be thinking about that anyway. We’re often there for a long time and they’ve fully recovered, but it’s nice to be able to leave something behind so that they can reference it later on. So, yeah, because it bridges that gap, I think it’s very useful.” AS3

Similarly, PWD found the booklet easy to read and understand and a good summary of advice and information:

“It is clear, and I would think if English weren’t your first language, you’d still be able to manage that. And that’s always something I take account of, could somebody who’s speaking English as a second or tenth language be able to access, and I’d say yes, they could.” P1

“It’s to the point, it’s not too much to take in [ … .] I found the advice very good and it’s what I do myself anyway.” P5

“ … .it’s very short and informative. It wants people to read it, doesn’t it, the way it’s presented.” P3

One PWD also discussed aspects of the booklet that they were not happy with, such as feeling it included incorrect or outdated information and was not appropriate for PWD on pumps:

“It’s not taking account of how diabetes and management technologies are changing at an exponential rate, really exponential. [ … .] more than 20% of us, and certainly more than 25% of us in Lincolnshire, don’t test our blood glucose very much anymore because we wear a continuous glucose sensor, so that’s out of date. [ … .] ‘Do not drive or operate machinery for at least 12 hours...’. The DBI rule is four hours.” P1

“I looked at it and I thought, ‘Well, it doesn’t even apply to me that’, because that’s for people who are on — you know we use the term MDI for the way most people manage their diabetes — but I have a pump, so we don’t follow MDI protocols, because they’re not appropriate. So, the leaflet didn’t even, you know, there’s a leaflet being dished out that doesn’t even cover people on pumps. And there are now quite a lot of us.” P1

#### Context

Ambulance staff and PWD felt that the success of a booklet intervention depended on many factors, especially PWD’ attitudes, motivation, and their willingness to assume the responsibility for managing their condition:

“ … there has to be some responsibility from the patient initially, and there to be an acknowledgement that they’ve got a disease that’s not going to go away, and they have to manage it, and it’s got to come from them. It can’t rest solely with EMAS.” AS2

“ … .it really depends on the individual and their attitude and motivation.” P3

A paramedic interviewee also felt that being an experienced professional can help overcome any potential implementation-related difficulties:

“Obviously, I’m an experienced paramedic, so there are hurdles and obstacles that we face every day really. And you overcome them, and once you overcome the first one, I think you just flow with it.” AS1

In addition, organisational culture was another factor that staff felt contributed to or detracted from the success of the intervention:

“We’re always encouraged by ours Comms, our direct managers, our clinical operation managers that we have at our stations. They’re always encouraging us to use these, to promote them, yes.” AS2

“I do think that it [EMAS’s patient safety and improvement culture] has changed [as a result of this study] and changed for the better.” AS1

Nonetheless, participants also identified various barriers to implementation of the ‘Hypos can strike twice’ booklet intervention. For staff members, the main barrier was that booklets were not always readily available:

“Many times when I wished to give these leaflets out there weren't any on the ambulance.” ASFTR6

“We don’t always have the leaflets available, whether it’s, you know, we’ve not had a chance to check they’re on the ambulance in the first place in the morning or we got sent straight out or there are none available on the station … ” AS4

Another barrier mentioned by staff was trying to implement the intervention with PWD who were reluctant to accept the booklet:

“Just when the patients weren’t that keen to participate or be forthcoming with information.” AS4

“It was just, I think, with the couple of problems I had, they were newer diabetics, so it was a bit of reluctance to take on that medical need really, I think.” AS1

A challenge mentioned by one staff member was being unable to reach the PWD’ doctor to complete patient safety-netting:

“The doctor wasn’t available; they didn’t answer their emergency phone basically [ … .] It was just to complete the safety-netting and leave at home by contacting the doctor also, but they weren’t as available as they should’ve been.” AS3

For PWD, one barrier to implementation would be PWD who were newly diagnosed or with accompanying issues, such as mental health issues, as it would be harder for them to cope with managing their condition:

“Did you know that a person with type one diabetes, it’s estimated, has to make 180 extra decisions a day in comparison with somebody who doesn’t have diabetes? … I think for newly diagnosed people, you know, it is hard. Now, you know, somebody newly diagnosed would probably find that leaflet an awful lot more use than somebody who has been diagnosed for as long as me.” P1

PWD also expressed their concern that a booklet might not be sufficient to affect change in behaviour:

“And if I’m honest, what’s my view of a leaflet? I think a leaflet is — by the time you’ve already had the hypo and I guess it depends on why people are having the hypo — a leaflet feels little lightweight to give somebody who’s had a hypo. [ … .] I guess it depends why people are going to hospital. Like I said, if it’s a recurring problem, some people I think should be referred to their diabetic specialist. A leaflet doesn’t feel appropriate to me. And who takes notice of a leaflet? But that’s just my view. I think a medical professional should be talking to somebody. I think it gives it more significance.” P2

Staff had a variety of attitudes towards booklets, with some feeling, as certain PWD did, that booklets might not be enough to change behaviour:

“I believe leaflets will not alter any mindset or behaviours. Those at risk of hypos already received professional literature from other agencies, most of which is at the bottom of a drawer somewhere. One patient I attended was given a leaflet but confessed to never reading it and threw it out.” ASFTR7

“I think, to be honest, the embarrassment of having an ambulance turn up on your doorstep for some people is enough to make them think, “Gosh, I better not let this happen again”. So, I think that is probably enough. I don’t know whether just a leaflet would promote that on its own.” AS2

Other staff felt that, despite some initial scepticism, booklets could affect behaviour change:

“Well, initially I didn’t think it would make a great difference, but then when I realised how much it was prompting me to talk about different things with the patients and their general lack of understanding of their condition and how they can prevent hypos, then I realised it would be more beneficial than I initially thought it was.” AS4

This positive attitude towards booklet interventions was further supported by staff members who also felt that research, especially in quality improvement, has helped advance care and improve outcomes for patients:

“Yeah, we’ve actually got a colleague who’s recently been diagnosed as a diabetic. She’s quite young and she’s got this in her arm now, but she’s also got a sensor on it that alerts her phone when she’s actually going into a hypo, or going towards, down to a hypo, so she knows when she’s got to take stuff on, you know. But I think all that has come from research and, you know, on the back of qualitative stuff, because how can you look into how things have been affected if you don’t do quality research on certain subjects and outcomes, you know.” AS1

#### Outcomes

Ambulance staff stated that implementing the HS2 intervention made them more aware that repeat hypoglycaemic events can happen often:

“I suppose it’s really hit me more whilst I was doing the study to realise that, you know, hypos can hit twice. [ … .] I still did what I needed to do to get them to a recovery stage. But did I think as much as I do now about them? I don’t think I did. So, you know, I think it’s important that obviously you’re aware that hypos can strike twice.” AS1

When asked about the benefits of the intervention for PWD, staff often reported being uncertain about its outcomes, as normally crew members do not receive any feedback on the progress of the PWD after leaving the scene:

“The only sceptical part of it was whether, when we referred the patients, did it actually get followed through. And, as I said to you earlier, we don’t have that information come back to us so we’re not sure — that is the only thing that I wasn’t happy about. There must be a way that our research department can look at that and just let us know … .” AS1

Despite that, most staff interviewed felt that the HS2 booklet intervention had been beneficial to PWD:

“Obviously, it’s hard for me to say what happens when I leave, what they do with the information, and the effect on further hypos with the patients, but it sounds like they’ve been receptive to the advice in general and they’ve taken it on board.” AS4

“I think that there will be instances where someone will have a second one and the leaflet won’t make any difference, but there will also be people that the leaflet prevents from having a second one if that makes sense. So, I don’t think it’s fool proof, but nothing will be, we’re dealing with the Great British public. There will always be problems, people who don’t take on the responsibility to manage their own condition and then have it happen again. But I think it maximises the chances of people recovering and not going the same way again, on that episode at least.” AS3

They also noted that they had not attended the same PWD twice for a repeat hypoglycaemic event, providing further evidence, in their mind, that the booklet had been beneficial:

“Yes, I think there must be something happening with them because, yeah, I only thought of that earlier on because I must have been to hundreds and hundreds of diabetics, [inaudible] and I must have been to hundreds over the years, and certainly over the last few years, I can’t remember going to an address where I’ve seen one of these leaflets. So, for me, that says that there must be something positive coming out of them because people are looking after themselves better. You know, maybe they’re looking at the warning signs a little bit better. I’m not saying it’s just down to the leaflet entirely, but maybe it has sparked some thoughts about what they could do to prevent getting a hypo before it gets too late.” AS2

More specifically, staff felt that the booklet provided PWD with all the necessary advice and information they need to prevent a second hypoglycaemic event:

“Very likely [the leaflet will reduce repeat ambulance attendances for hypoglycaemic events] if the patients and the people that you inform take on the advice given, yeah, because I think a lot of the cases that I’ve been to, the hypos, you can find a cause for it, it’s preventable. So, they might have had an infection, or they’ve not eaten that day, or they’ve taken the wrong dose of insulin. Most of them are preventable with education on how to manage them better.” AS4

“Yeah, so I think because with the leaflet you’re spending more time on scene, so you’re giving that extra knowledge to the patients. Because I don’t think a lot of them realise that they could have a second hypo. And one of the questions we ask routinely now is, “Once you’ve had a hypo, do you happen to have another hypo within a few hours?” At the time, it was going by the leaflet, so I think it was informing them well.” AS1

The two PWD who remembered receiving a booklet had different views on whether the booklet had been beneficial. One PWD felt that booklets cannot make a difference, whereas the second PWD felt that the booklet had been beneficial, mainly as a reminder to be more careful:

“No, a leaflet won’t make any difference, you know, because people will either do it or they won’t. [ … .] A leaflet, no, it’s just kind of not on the radar really.” P1

“Yes, it did. It did help. But I’ve had to be independent because I live alone, like so many people. [ … .] Just take extra care.” P5

PWD who had not received (or could not remember receiving) a booklet felt that they were already aware of the information and advice included in the booklet, so most likely it would not have made a difference to them:

“Not for me in particular as I nursed for a while so I knew, and I’ve been dealing with my own insulin for a little while, so in many ways it wouldn’t have worried me not to have it.” P4

“I don’t remember learning anything new from the leaflet” P3

#### Suggestions for improvement

Participants had various ideas on how to improve the HS2 intervention. Ambulance staff members, for example, suggested including more information about local support services available to PWD, or even adding a verbal clinician to clinician safety net:

“I think the only thing you could probably do is put more in there about local services that are available dependent on location — might be a good thing.” AS2

“Free handwriting for further treatments, options available for useful contacts.” ASFTR 8

“I personally feel a verbal clinician to clinician safety net, such as local diabetic pathways, more beneficial as it guarantees a HCP [healthcare professional] follow up and more tailored care plan.” ASFTR9

A similar suggestion, signposting PWD to other useful resources, was given by a PWD as well:

“ … . that’s where I would go to initially [referring to the British Diabetic Association website], so maybe a leaflet that highlights that there is a very well-established association with lots of information, because they do fact sheets.” P3

Both ambulance staff and PWD also suggested creating different types of booklets, based on the characteristics of the PWD, such as type of diabetes, the medication they are on, type of monitoring device, their age, etc.:

“Yes, I think especially for type 1s, a separate one for type 2s. Some that have tablets only and some that have insulin and tablets as well. Specific ones for specific age categories would be … yeah that sounds a good idea actually. You could have your young diabetics as well, learning to come to grips with having to deal with it for the rest of their lives. I mean, it’s going to affect everything. But at the moment, I think there’s a nice balance in there of information, because I think sometimes too much information can be overwhelming. For the ones that have been dealing with it for years, possibly a smaller version of it. I don’t know, but yeah, that’s a good idea, possibly.” AS2

“Right, two things: one, it needs a different leaflet for people who use a pump; and two, both the leaflet for pumpers and MDIers needs to acknowledge that we use flash and continuous glucose monitors, because we may be a minority, but we are an increasingly large minority.” P1

“ … . things have got to be geared towards individuals. It’s not that everybody needs the same.” P3

A PWD also suggested replacing the booklet with an app, offering advice and information depending on the type of hypoglycaemic event they were experiencing:

“Well, you know, you could just have an app called something like ‘Hypoaware’ that would have on it strategies for dealing with different types of hypo. Because the other thing is, they can be ever so different. You know, you might sort of end up very confused in one sort of hypo; you might end up very, very depressed, and just end up crying in another hypo. So, an app that helped you to recognise what type of hypo it was before you ended up on the floor. Or one that your carers or whoever found you would be able to access.” P1

Finally, one crew member was so satisfied with the HS2 booklet that they suggested creating and distributing more booklets for different conditions:

“ … . But using it, and seeing it used, putting it into practice and seeing its effect, now I think it’s perfect and I think we could do with lots of others.” AS3

#### Integration of quantitative and qualitative results

A summary of the survey and interview data from both PWD and staff can be seen in the joint display (Table 4).

**Table 4:** Joint display summarising survey and interview data from PWD and staff

Process evaluation components		Survey data		Interview data	
		PWD data	Staff data	PWD data	Staff data
**Implementation**	Implementation process & delivery	• 29.3%^a^ reported receiving the booklet	• 22.5% often & 28.8% always gave out the booklet to eligible PWD	2 out of 5 interviewed PWD had received or remembered having received a booklet	• Successfully implemented the intervention with the majority of eligible PWD • Some were never told about the booklets/intervention
	Fidelity	No relevant data.	• 46.3% always completed all sections of the booklet	No relevant data.	• Implemented as instructed to
	Training	No relevant data.	10.0% of staff reported receiving training. All agreed that the training was timely, relevant, and sufficient	No relevant data.	Timely and relevant Most did not receive training
	Resources	No relevant data.	78.0% did not need any support implementing the intervention 72.9% felt that colleagues could help with and 74.6% that colleagues could answer questions about the intervention, if needed	No relevant data.	Could rely on a colleague/contact researcher for guidance
**Mechanisms of impact**	Reception of the intervention	66.6% were happy to receive the booklet and the extra advice they were given	49.2% felt that PWD found the intervention helpful	Felt the booklet would be more useful to someone with less experience or who cannot manage their diabetes well	Most were receptive, but some not as much (booklet was extra work) Felt that most PWD were receptive and found it useful, but for some it was too much to read Very experienced & newly diagnosed PWD, who were in denial, were less receptive
	Acceptability of the intervention	50.0% felt there was a need for such an intervention & 75.0% that the booklet did meet their needs 80% felt it was easy to understand how to use the booklet 80.0% felt it was easy to follow the advice given by the ambulance staff 75.0% felt that the booklet added value to the care they received	67.2% said there was a need for such an intervention & 63.1% that the booklet met the needs of the PWD 33.8% found the intervention easy to implement & 15.6% very easy to implement (neutral = 37.7%) 44.30% were very confident & 11.4% extremely confident implementing it 75.4% felt that participating in the intervention fitted well with their existing work 69.2% felt that delivering this intervention added value to attending to the PWD	Booklet was easy to read/understand Booklet was a good summary of advice and information One PWD felt the booklet included incorrect or outdated information and was inappropriate for PWD on pumps	There was a need for such an intervention (safety netting & reminder for PWD) Easy to implement Fits well with existing role & workload Enhanced or complemented care Booklet was easy to read and understand, informative, useful as a record of care, an addition to or continuation of care
	Experience with the intervention	41.6% felt that being given this booklet had made things easier for them	No relevant data.	Most didn’t learn anything new from booklet Potentially more useful to someone with less experience	Overall positive
**Context**	Barriers to implementation	No relevant data.	No relevant data.	For newly diagnosed PWD or those with accompanying issues it is harder to cope with managing diabetes	Booklets were not always readily available Implementing the intervention with PWD who were reluctant to accept the booklet Being unable to reach physicians to complete patient safety-netting
	Facilitators to intervention	No relevant data.	No relevant data.	The attitudes of PWD, their motivation, & willingness to assume responsibility for managing diabetes	The attitudes of PWD, their motivation, & willingness to assume responsibility for managing diabetes Being an experienced professional helps overcome potential implementation-related difficulties
	Attitudes	63.7% felt that booklets have the ability to change everyday behaviour of PWD	71.9% felt that implementing the HS2 booklet had improved their feeling that quality improvement interventions are helpful for PWD	Booklets are not appropriate or sufficient to affect change in patient behaviour	Some felt booklets are not enough to change patient behaviours Others felt booklets can indeed affect change in patient behaviours Research can help advance care and improve outcomes for patients
	Organisational culture	No relevant data.	Staff members felt that their organisation’s culture of safe care (66.7%) and improvement culture (61.7%) supported the implementation of the intervention Staff members felt that implementing this intervention had also affected their organisation’s patient safety (16.67%) and quality improvement (20.0%) culture	No relevant data.	Organisational culture contributed positively to the success of the intervention and vice versa
**Outcomes**	Changed patient behaviours	50.0% found the intervention to be beneficial (neutral = 41.7%) Receiving the booklet reminded/encouraged: ▪ 18.9% to have a chat/check up with their GP ▪ 45.5% to have a chat/check up with their diabetes consultant ▪ 20% to adjust their medication ▪ 54.6% to test their blood glucose more often ▪ 45.5% to adjust their diet ▪ 27.3% to avoid alcohol for 24 h ▪ 30.0% to reduce their overall alcohol consumption ▪ 90.0% to exercise more ▪ 20.0^a^% to not drive or operate machinery for at least 12 h ▪ 30% to avoid strenuous activity for 24 h. 30% felt that having received the booklet, their behaviour had changed for the better 77.7% also felt that having received advice from the ambulance staff, had changed their behaviour for the better	87.93% felt that the intervention had been beneficial to PWD 60.3% felt that the intervention might enhance self-care	One PWD felt that booklets cannot make a difference. One PWD felt that the booklet had been beneficial, mainly as a reminder to be more careful. PWD who had not received (or could not remember receiving) a booklet felt that they were already aware of the information and advice included in the booklet, so most likely it would not have made a difference to them	Most felt that the intervention had been beneficial to PWD
	Prevention of second hypo	36.3% felt that having the HS2 booklet might prevent recurrent hypoglycaemia episodes (neutral = 63.7%)	41.4% felt that the intervention might prevent recurrent hypoglycaemia episodes	No relevant data.	Uncertain about its outcomes (crew members do not receive any feedback on the progress of the PWD after leaving the scene)
	Reduction of repeat ambulance attendances for hypos	No relevant data.	41.4% felt that the intervention might reduce repeat ambulance attendances for hypoglycaemia episodes	No relevant data.	Never had to attend to the same PWD twice for a repeat hypoglycaemic event
	Other	No relevant data.	No relevant data.	No relevant data.	Staff now more aware that repeat hypoglycaemic events can happen often

^a^Numbers represent the percentage of survey participants who responded to each relevant item/question

#### Implementation

Most ambulance staff members reported implementing the booklet intervention to all eligible PWD with diabetes experiencing a hypoglycaemic event. However, only a third of the PWD participants said they had received (or remembered receiving) the HS2 booklet.

Most ambulance staff reported not having received any training on the intervention (those who did, found the training timely and relevant), but also felt they did not need any support while implementing it and there was often a colleague they could turn to for further guidance.

#### Mechanisms of impact

The intervention was generally well received by both ambulance staff members and PWD, although staff members felt that very experienced and newly diagnosed PWD, who were in denial, were less receptive.

Ambulance staff also felt the intervention was easy to implement and felt confident implementing it. They also felt that participating in the intervention fitted well with their existing workload and it enhanced or complemented the care they provided. PWD also felt that the booklet and the advice they received from the ambulance staff members added value to the care they received. In addition, both staff and PWD felt the booklet was easy to read and understand and that it was a good summary of advice and information. One PWD we interviewed, though, felt that the booklet included incorrect or outdated information and was inappropriate for PWD on pumps.

Most participants felt there was a need for such an intervention and that the booklet met the needs of the PWD. Although our survey results showed that most PWD and staff members felt that the intervention was useful and beneficial for PWD, our qualitative findings painted a different picture. The PWD we interviewed were all very experienced at managing their diabetes and had had a diagnosis for many years. As a result, they felt that they didn’t learn anything new from the booklet (although some found it a useful reminder) and felt that it would be more valuable to someone with less experience or who cannot manage their diabetes well.

#### Context

The main barriers to implementation for ambulance staff members were implementing the intervention with PWD who were reluctant to accept the booklet, sometimes being unable to reach physicians to complete patient safety-netting, and not always having booklets readily available at their vehicles and/or stations. PWD felt that a possible barrier would be PWD who are newly diagnosed or with accompanying issues, as it is harder for those PWD to cope with managing their diabetes.

On the contrary, participants felt that the positive attitudes of PWD, their motivation, and willingness to assume responsibility for managing diabetes were the main facilitators to receiving and benefiting from the HS2 intervention. In addition, ambulance staff members felt that being an experienced professional can help overcome potential implementation-related difficulties.

Regarding the attitudes of the PWD towards booklets and quality improvement interventions in general, we found that changing their behaviour can be challenging, but PWD felt that booklets had the ability to change behaviours. According to the PWD survey results, some of the lowest scores corresponded to the questions enquiring about the change in eating, sleeping, exercising behaviour (Q19-Q23), with most PWD disagreeing or neither agreeing nor disagreeing with it. Our interview findings show that PWD believed that booklets are not appropriate or sufficient to affect change in behaviour (especially for something as serious as a hypoglycaemic event). This could potentially be attributed to the fact that the PWD we interviewed were all experienced at managing their diabetes and felt they had not learned any new information from the booklet. Ambulance staff members had various views, with some feeling that booklets are not enough to change behaviours (results from interviews), whereas others felt that booklets and similar interventions can indeed affect change in behaviours (both quantitative and qualitative findings).

Ambulance crew members also felt that research can help advance care and improve outcomes for patients and that their organisation’s (EMAS’) patient safety and improvement culture contributed positively to the success of the intervention (and vice versa).

#### Outcomes

Ambulance staff members felt that the booklet intervention had made them more aware of the possibility of repeated hypoglycaemic events and had also been beneficial for PWD. This was evident from both quantitative and qualitative data. Half of the PWD that participated in the survey also found the intervention to be beneficial (with another 41.7% being neutral), but we only interviewed two PWD who remembered receiving the booklet: one of them felt that booklets cannot make a difference, whereas the second one found the booklet to be beneficial, mainly as a reminder to be more careful. Those who could not remember receiving a booklet felt that they were already aware of the information and advice included in the booklet, so most likely it would not have made a difference to them.

When asked about the potential of the booklet/intervention preventing a second hypoglycaemic event or reducing the number of repeat ambulance attendances, ambulance staff members who were interviewed were uncertain as they did not normally receive feedback on the progress of the PWD after leaving the scene. They did, however, report never having to attend to the same PWD twice for a repeat hypoglycaemic event. According to their survey answers, there were slightly more staff members who felt that the HS2 intervention may prevent a second hypoglycaemic event (37.93% vs 27.59%) and/or may reduce the number of repeat ambulance attendances for such an event (36.21% vs 27.59%). Results from the PWD survey showed that PWD were less certain about the effects of the intervention on preventing a second hypoglycaemic event (63.7% were neutral), but also showed that receiving the booklet reminded and/or encouraged them to test their blood glucose more often, adjust their diet, and have a chat/check up with their diabetes consultant.

Finally, ambulance staff members planned to continue implementing the intervention and handing out the HS2 booklets to eligible PWD and felt that the intervention could be implemented successfully in other ambulance services as well.

## Discussion

### Main findings

This process evaluation for the ‘Hypos may strike twice (HS2)’ intervention, which was found to lead to a significant reduction in repeat ambulance calls for hypoglycaemia [11], revealed positive attitudes to the intervention from both ambulance staff and PWD. The intervention was not implemented in all cases of hypoglycaemia, with staff survey responders expressing they used it in half the cases; interviewed staff, though, reported implementing it to almost all eligible PWD. Similarly, only a third of PWD survey responders, and 2 out of 5 interviewed PWD, remembered receiving the HS2 booklet. Most staff members considered that the booklet was informative, easy to use and to explain. Moreover, they were not concerned that the booklet could hinder their work or staff-patient interaction. PWD also showed positive attitudes, but those we interviewed felt that the booklet intervention would be more valuable to less experienced PWD or those who cannot manage their diabetes well. This could potentially be attributed to the fact that these PWD were all experienced at managing their diabetes and felt they had not learned any new information from the booklet. Overall, participants felt that the intervention can be beneficial to PWD (especially as a reminder to be careful and adjust their behaviour accordingly) but were uncertain about whether it can help prevent a second hypoglycaemic event and/or reduce the number of repeat ambulance attendances. These results should be interpreted cautiously since responses to surveys and interviews were low.

### Strengths and limitations

Despite the fact that the quantitative analysis showed that female ambulance staff were less optimistic about the booklet’s future effects (compared to male staff), we were able to recruit and interview mainly male staff members (3/4). The qualitative findings of ambulance staff should, therefore, be interpreted with caution and further research with a more diverse population should be conducted to explore these findings in more detail. The findings were also limited by overall low numbers of responders to surveys and interviews. Recruitment happened as the Covid 19 pandemic was unfolding and this may have affected recruitment numbers. This was mitigated to some extent by the strengths of the study, such as the use of mixed methods with both self-administered surveys and interviews of PWD and staff, and integration of data across these. Conducting the interviews online may have been another redeeming factor.

### Interpretation

Ambulance services have been responding to PWD and severe hypoglycaemia for over 40 years [15] and in the past two decades, clinicians have been safely treating and discharging them without conveying to hospital [16–18]. Ambulance attendance for severe hypoglycaemia leading to hospitalisation is not only common and costly [4, 5], but in type 2 diabetes, hypoglycaemia involving an ambulance call has been associated with greater mortality [19].

Reattendance for recurrent hypoglycaemia is also common, affecting 5% within 48 h [20] and 10% within 3 months [21]. Current UK guidelines for ambulance services have advocated communicating with primary care or diabetes nurse teams following an episode of hypoglycaemia [22] but despite this recommendation, follow-up, therapy change or specialist intervention in people self-reporting severe hypoglycaemia are uncommon, suggesting that care pathways are poorly developed or not used [21, 23].

Booklets have been found to improve patient knowledge [8, 9, 24] and satisfaction [24], particularly in acute conditions where information may be lacking [24]. In the Emergency Department setting they have also been found to improve communication, change clinical behaviour, and reduce emergency reattendances for the same condition [25].

Ambulance pathways for hypoglycaemia involving contact from a specialist team are being implemented and tested [23, 26] because direct paramedic referral [27] or self-referral [28] has not always been found effective, but this (HS2) intervention was found to demonstrably reduce repeat attendances [11].

### Implications for future practice, policy, and research

Because of its simplicity, low cost, and effectiveness [11], we advocate HS2 for wider use by ambulance services. The benefits should be maintained and spread to other services, but the effects of the intervention may also be enhanced by incorporation in behavioural theory-informed complex interventions and inclusion of direct referrals to community pathways. These interventions should be tested and evaluated further [11].

### Recommendations

Based on the findings from both staff and PWD surveys and interviews, the following recommendations are made for ambulance services that may implement booklet-based interventions in the future:

- All staff need to be informed about the availability of the booklet and its purpose, as well as available training on implementing the intervention.

- Measures should be put in place so the booklets are readily available for use in stations and emergency vehicles.

- Staff need to be encouraged to always provide PWD with the booklet regardless of their age or their history of diabetes, since PWD who had just been diagnosed may find the information new and useful and PWD with long history of the condition may find the information a good reminder.

## Conclusion

The ‘Hypos may strike twice’ intervention was found to be feasible, acceptable to PWD and staff, prompting reported behaviour change and help-seeking from primary care, with demonstrable reductions in repeat attendances. The findings of this process evaluation will help wider adoption of the intervention.

## Supplementary Information

**Additional file 1.** “Hypos can strike twice” booklet.

**Additional file 2.** Supplementary materials and results.

## Data Availability

The datasets used and analysed during the current study are available from the corresponding author on reasonable request.

## Abbreviations

AS: Ambulance staff
EMAS: East Midlands Ambulance Service NHS Trust
EMT: Emergency Medical Technician
FTR: Free text responses
GP: General practitioner
HCP: Health care professional
HS2: Hypos can strike twice intervention
IQR: Interquartile range
MDI: Multiple daily injections
MRC: Medical Research Council
P: Person with diabetes
PWD: People or person with diabetes
Q: Question(s)
UK: United Kingdom
