# Supplementary material for: People with diabetes and ambulance staff perceptions of a booklet-based intervention for diabetic hypoglycaemia, “Hypos can strike twice”: a mixed methods process evaluation
Source: BMC Emerg Med. 2022 Feb 8;22:21. doi: 10.1186/s12873-022-00583-y (PMC8822761; doi:10.1186/s12873-022-00583-y)
Supplement: Supplementary file 1 — Additional file 1. “Hypos can strike twice” booklet. [file 12873_2022_583_MOESM1_ESM.pdf]

NHS
East Midlands
Ambulance Service
NHS Trust

‘HYPOS’ can strike twice

A GUIDE FOR PEOPLE WITH DIABETES WHO HAVE HAD A HYPOGLYCAEMIC (‘HYPO’) EVENT

- *What is a ‘hypo’?*
- *Who has ‘hypos’?*
- *How to treat a ‘hypo’?*
- *How to avoid a ‘hypo’?*

East Midlands Ambulance Service (EMAS) responded to a 999 call to treat you for a 'hypo'

Name:	DOB:
Address:	Job No:
	GP Surgery:
Location: ☐ Home ☐ Other (please specify)	Lives Alone: Yes ☐ No ☐
Date & Time of EMAS arrival:	
Time of EMAS departure:	

How you were	When we arrived	EMAS intervention	When we left
Time of reading (hh/mm)			
Breathing rate			
Blood glucose (mmol/l)			
Oxygen saturations			
Heart rate			
Blood pressure (mm/Hg)			
Temp (°C)			
ECG rhythm (12 lead)			
Pain score	/10		/10
Conscious level (please tick)	ALERT ☐<br>VERBAL ☐<br>PAIN ☐<br>UNRESPONSIVE ☐		ALERT ☐

**Treated with: Fast acting carbohydrate** ☐ **IV Glucose** ☐	**GlucoGel®** ☐ **IM Glucagon®** ☐
**Conveyed:** ☐ **No** ☐ **Yes (specify):**	
**Clinician Referral to:**	

You have had a ‘hypo’ and were treated with fast-acting carbohydrate:

You were conscious and able to swallow but unable to treat yourself so you were treated with fast acting carbohydrates: (EMAS please ✔)

- ☐ 60 mls Glucojuice®
- ☐ 4 large or 7 small jelly babies
- ☐ 150 ml - 200 ml of smooth orange juice
- ☐ 5 - 6 GlucoTabs®
- ☐ 5 - 6 Dextrose® tablets

Other:

You were conscious but unable to treat yourself, but still able to swallow so you were treated with: (EMAS please ✔)

- ☐ 25g GlucoGel®

You were unconscious and treated with: (EMAS please ✔)

- ☐ 1mg Glucagon® intra-muscularly
- ☐ 10% intravenous glucose

You will be referred to your local diabetes specialist nurse team or your GP. You may be contacted and asked about your current medication and advised about how to prevent another episode.

Please telephone your GP surgery if you do not want to be contacted.

Now you have recovered:

- If you are not due to eat a meal, eat some starchy food like a sandwich or a banana
- You should eat regularly over the next 24 hours, including starchy food e.g. rice, bread or potato
- You may need to reduce your insulin dose or your diabetes medication - you must do this in discussion with your GP, Diabetes Health Professional or, at any other time, through the Out of Hours GP
- If you have a meter, test your blood glucose every 4 hours during waking hours
- Do not drive or operate machinery for at least 12 hours
- Avoid strenuous activity for 24 hours
- Avoid alcohol for 24 hours

Your diabetes medication:

Please tell us your current diabetes medication. This may help us to understand why you have had a 'hypo'.

EMAS PLEASE COMPLETE

Frequently asked questions:

What is a 'hypo'?

Glucose is a sugar carried in the bloodstream, which your body uses for energy. If you have diabetes, your blood glucose levels can go up and down, sometimes becoming very low – this is called hypoglycaemia (or a 'hypo'), and this happens when your blood glucose goes below 4 mmol/l.

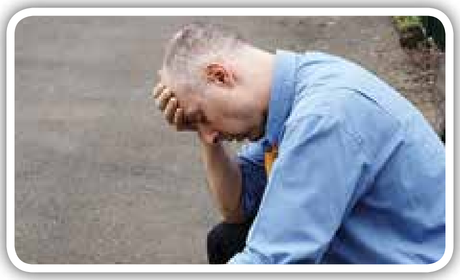

Symptoms

Early signs and symptoms of a 'hypo' include:

- Sweating heavily
- Feeling anxious
- Trembling and shaking
- Tingling of the lips
- Hunger
- Going pale
- Palpitations

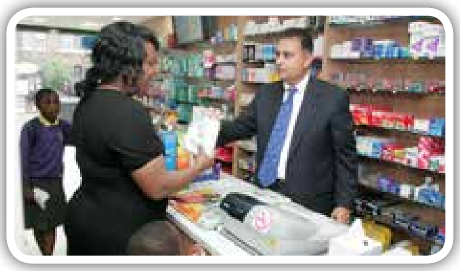

Symptoms can vary from person to person, but you will feel "unwell" very quickly. If you miss these early signs, the symptoms may get worse and lead to:

- Slurring of your words
- Behaving oddly
- Being unusually aggressive or tearful
- Having difficulty in concentrating

If you do not treat your 'hypo' at this stage, you may become unconscious.

Some people may not get any warning signs.

‘Hypo’: Who is at risk of a ‘hypo’ and why?

Who has ‘hypos’?

- If you are injecting insulin or taking diabetes tablets that make your body produce more insulin, then you may be at risk of ‘hypos’
- If you are not sure how your diabetes tablets work, discuss this with your local pharmacist or your Diabetes Heath Professional when they contact you.

What causes ‘hypos’?

A number of situations can cause a ‘hypo’:

Drinking too much alcohol or drinking alcohol without eating

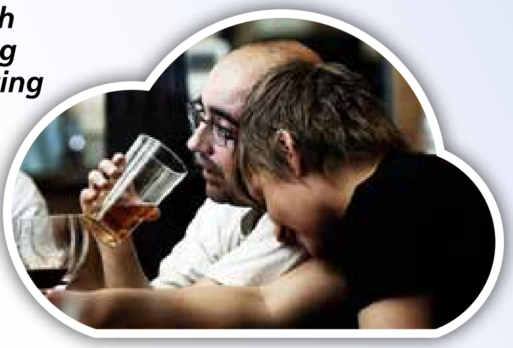

Eating less starchy foods than usual

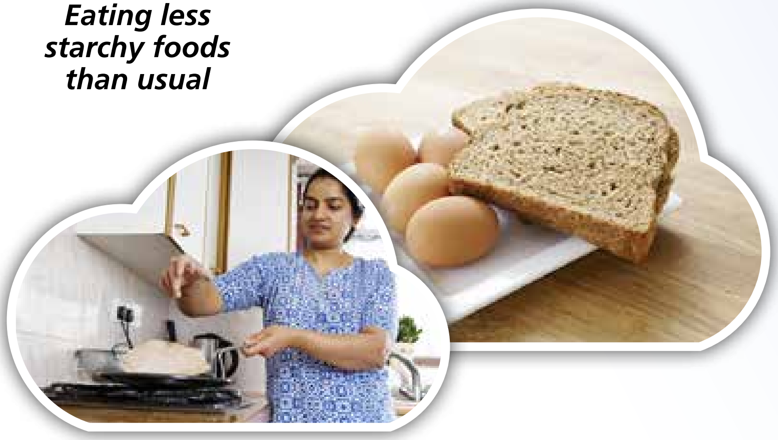

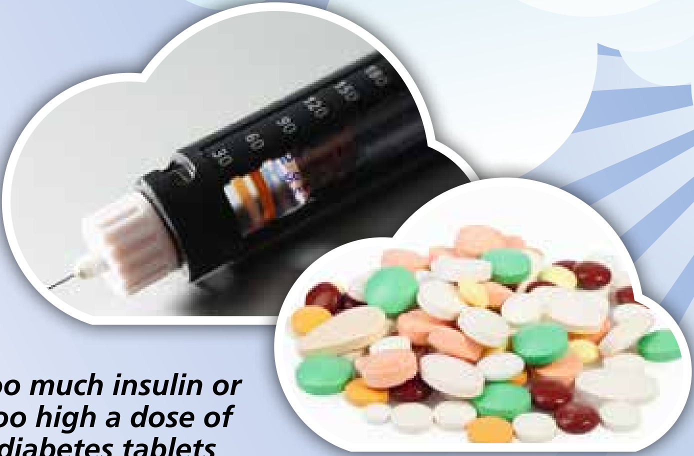

Too much insulin or too high a dose of diabetes tablets

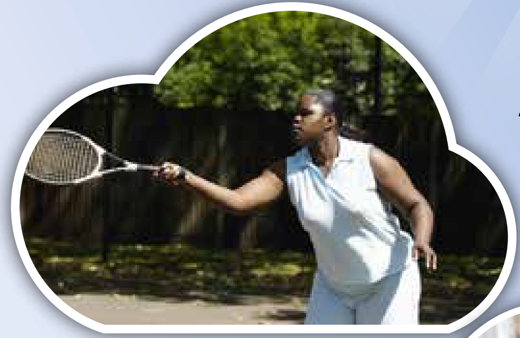

Unplanned or strenuous activity

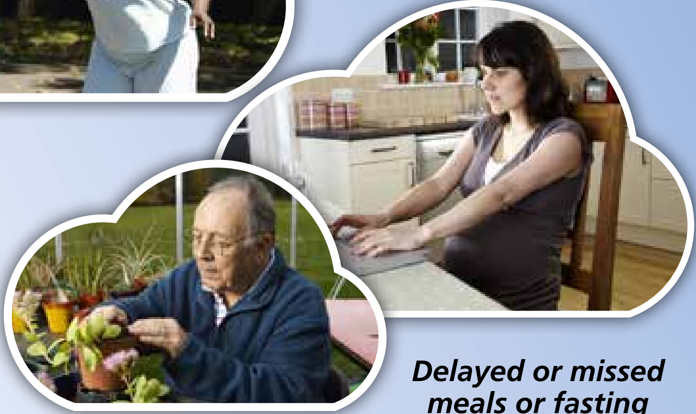

Delayed or missed meals or fasting

‘Hypo’: Who is at risk of a ‘hypo’ and why?

Sometimes there is no obvious cause, but treatment should always be carried out immediately with food or drink that will raise your blood glucose quickly, as per the following advice.

Suitable initial treatments for ‘hypos’ are:

- **60 mls Glucojuice®**
- **4 large or 7 small jelly babies**
- **25g GlucoGel®**
- **150ml - 200 ml (a small carton) of smooth orange juice**
- **5 - 6 GlucoTabs®**
- **5 - 6 Dextrose tablets**

IMPORTANT!

- **If you do not feel better or your blood glucose level is still less than 4 mmol/l after 10 to 15 minutes, repeat ONE of these treatments.**
- **If you are no better after THREE treatments seek healthcare professional advice**

If you need help treating your 'hypo'

If you are not able to treat your 'hypo' yourself, but are still conscious and able to swallow, someone can give you glucose gel if you have this available.

They should slowly squeeze the gel from one sachet into the inside of your cheek and around your gums. They should gently rub the outside of your cheek as this will help absorption into your gums. If done correctly it can take up to 15 minutes to use the whole sachet. You may need to be treated with two sachets. Once you are able to eat and drink, you should eat a starchy snack.

If you become unconscious, you need **immediate** emergency treatment. Someone should dial **999** for an ambulance and you should be put on your side with your head tilted back.

If you are unconscious, glucose treatments should NOT be put in your mouth.

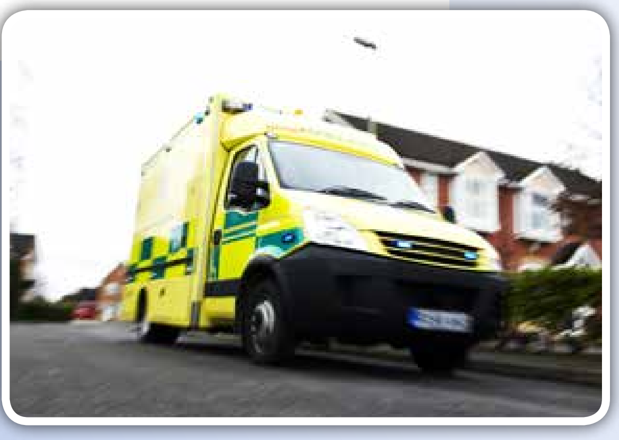

How to avoid 'hypos'

- Eat regularly
- You may need to eat more carbohydrates before and after physical activity
- Keep to sensible alcohol limits and do not drink on an empty stomach
- Take your medication at the recommended dose and times
- If you are testing your blood glucose levels, and notice your readings are regularly dropping, discuss this with your diabetes team as you may need a change in medication or to have your insulin adjusted
- Always carry glucose and a starchy snack with you to treat 'hypos' quickly

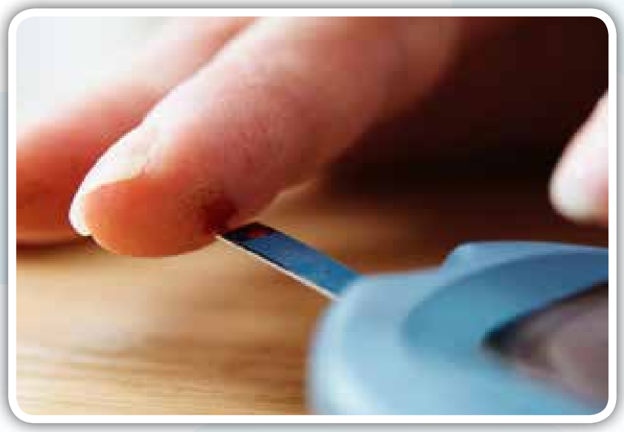

WARNING SIGNS

IMPORTANT

- Never ignore the warning signs of a ‘hypo’
- Act immediately by eating or drinking something that will raise your blood glucose quickly
- Make sure other people know what to do when you are having a ‘hypo’

Driving and diabetes

Having diabetes does not mean that you have to give up driving, but it does mean that you need to plan in advance before you get behind the wheel. Insulin and tablets called Sulphonylureas (e.g. Gliclazide, Glipizide and Glibenclamide) can put you at risk of 'hypos'.

Complications associated with diabetes and particularly with 'hypos' can lead to confusion which will affect your ability to drive. This can increase the risk of accidents and may, in turn, lead the DVLA to consider stopping you driving for a period of time.

Your Diabetes Healthcare Professional will be able to help you learn more about your own 'hypo' signs and symptoms so you can ensure continued safe driving.

IMPORTANT

Always carry glucose and starchy food, e.g. a cereal bar, in the car

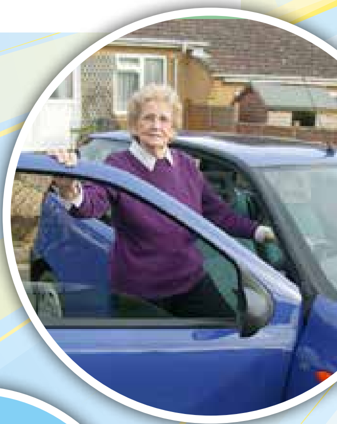

IMPORTANT

If you have a 'hypo' whilst driving, stop the car as soon as possible. Remove the keys to demonstrate you are not in charge of the vehicle and move into the passenger seat if it safe to do so. Treat the 'hypo' as advised in this booklet

IMPORTANT

Do not drive if your blood glucose is 5mmol/l or less. Eat and then retest

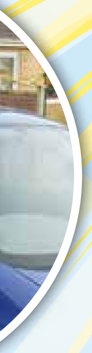

This document has been designed for people who have been diagnosed with diabetes and are recovering from a hypoglycaemic ('hypo') event.

If you need more information or further explanation contact your Diabetes Healthcare Professional to discuss your concerns.

Further Information

For further information about your local ambulance service, please contact:

East Midlands Ambulance Service NHS Trust

Trust Headquarters
1 Horizon Place
Mellors Way
Nottingham Business Park
Nottingham,
NG8 6PY

Call us: 0115 884 5000

Or visit us at: www.emas.nhs.uk

© East Midlands Ambulance Service (EMAS)

Original concept by:

East Midlands Ambulance Service (EMAS)

Specialist advice:

June James
University Hospitals of Leicester NHS Trust

Helen Atkins & Judith Spiers
University Hospitals of Leicester NHS Trust

Design by:

Michael Bonar & Shehnaz Jamal
Leicester Diabetes Centre
www.leicesterdiabetescentre.org.uk

Notes:

University Hospitals of Leicester NHS
NHS Trust

Leicester Diabetes Centre

Supported by NIHR

The development of this booklet was supported by the National Institute for Health Research's Collaboration for Leadership in Applied Health Research and Care East Midlands.

The views expressed are those of the author(s) and not necessarily those of the NHS, the NIHR or the Department of Health and Social Care.

@CLAHRC_EM | www.clahrc-em.nihr.ac.uk
