# Supplementary material for: People with diabetes and ambulance staff perceptions of a booklet-based intervention for diabetic hypoglycaemia, “Hypos can strike twice”: a mixed methods process evaluation
Source: BMC Emerg Med. 2022 Feb 8;22:21. doi: 10.1186/s12873-022-00583-y (PMC8822761; doi:10.1186/s12873-022-00583-y)
Supplement: Supplementary file 2 — Additional file 2. Supplementary materials and results. [file 12873_2022_583_MOESM2_ESM.docx]

**SUPPLEMENTARY MATERIALS AND RESULTS**

**Table S1 Ambulance staff survey characteristics**

| **Characteristic** | | **N** | **%** |
| --- | --- | --- | --- |
| **Sex** | Female | 26 | 45.6 |
|  | Male | 31 | 54.4 |
|  |  |  |  |
| **Age** | 18-24 | 12 | 21.1 |
|  | 25-34 | 21 | 36.8 |
|  | 35-44 | 10 | 17.5 |
|  | 45-54 | 13 | 22.8 |
|  | 55-64 | 1 | 1.8 |
|  |  |  |  |
| **Qualification** | Technician | 15 | 39.5 |
|  | Paramedic | 15 | 39.5 |
|  | ECA | 4 | 10.5 |
|  | EMT | 4 | 10.5 |
|  |  |  |  |
| **Years of experience** | under 1 year | 2 | 3.5 |
|  | 1-4 years | 26 | 45.6 |
|  | 5-9 years | 11 | 19.3 |
|  | 10-14 years | 2 | 3.5 |
|  | 15-19 years | 5 | 8.8 |
|  | Over 20 years | 11 | 19.3 |
|  |  |  |  |
| **History of diabetes** | Yes | 20 | 35.1 |
|  | No | 36 | 63.2 |
|  | Don’t know | 1 | 1.8 |

**Table S2 People with diabetes (PWD) survey responder characteristics**

| **Characteristic** | | **N** | **%** |
| --- | --- | --- | --- |
| **Sex** | Female | 20 | 43.5 |
|  | Male | 26 | 56.5 |
|  |  |  |  |
| **Age** | 16-17 | 1 | 2.2 |
|  | 18-24 | 0 | 0 |
|  | 25-34 | 2 | 4.4 |
|  | 35-44 | 3 | 6.5 |
|  | 45-54 | 6 | 13.0 |
|  | 55-64 | 9 | 19.6 |
|  | 65-74 | 14 | 30.4 |
|  | 75-84 | 6 | 13.0 |
|  | 85 or over | 5 | 10.9 |
|  |  |  |  |
| **Marital Status** | Single | 7 | 15.2 |
|  | Living with someone | 1 | 2.2 |
|  | Married | 32 | 69.6 |
|  | Widowed | 5 | 10.9 |
|  | Prefer not to say | 1 | 2.2 |
|  |  |  |  |
| **Ethnicity** | White | 39 | 86.7 |
|  | Mixed | 2 | 4.4 |
|  | BAME | 4 | 8.9 |
|  |  |  |  |
| **Diabetes type** | Type 1 | 24 | 54.6 |
|  | Type 2 | 19 | 43.2 |
|  | No answer | 1 | 2.3 |
|  |  |  |  |
| **Diabetes time** | 2-5 years | 3 | 6.8 |
|  | Over 5 years | 41 | 93.2 |
|  |  |  |  |
| **Hypos in last year** | 1-4 | 30 | 73.2 |
|  | 5-9 | 4 | 9.8 |
|  | 10-14 | 4 | 9.8 |
|  | 15-19 | 1 | 2.4 |
|  | 30-39 | 1 | 2.4 |
|  | 50 or over | 1 | 2.4 |
|  |  |  |  |
| **GP visits** | Once a month | 5 | 11.1 |
|  | Every other month | 2 | 4.4 |
|  | Every 3 months | 4 | 8.9 |
|  | Every 6 months | 11 | 24.4 |
|  | Once a year | 15 | 33.3 |
|  | Not sure | 8 | 17.8 |

**Table S3 Scoring of each questionnaire item of the Staff Survey. Medians/IQRs are shown in brackets.**

|  | | **Never** | | **Rarely** | | **Sometimes** | | **Often** | | **Always** | |
| --- | --- | --- | --- | --- | --- | --- | --- | --- | --- | --- | --- |
| 1. How often did you give out the leaflet to patients with hypos? | | 12  15% | | 13  16.25% | | 14  17.50% | | 18  22.50% | | 23  28.75% | |
|  | | **4 [2,5]** | | | | | | | | | |
| 2. How often did you complete all the sections of the leaflet? | | 15  18.75% | | 5  6.25% | | 8  10.00% | | 15  18.75% | | 37  46.25% | |
|  | | **4 [2.5, 5]** | | | | | | | | | |
|  | | **Very Difficult** | | **Slightly Difficult** | | **Neutral** | | **Easy** | | **Very Easy** | |
| 3. How easy/difficult was it to implement the HS2? | | 3  3.90% | | 7  9.10% | | 29  37.66% | | 26  33.77% | | 12  15.58% | |
|  | | **3 [3,4]** | | | | | | | | | |
|  | | **Not at all** | | **Slightly** | | **Moderately** | | **Very** | | **Extremely** | |
| 4. How confident were you implementing the intervention? | | 9  11.39% | | 8  10.13% | | 18  22.78% | | 35  44.30% | | 9  11.39% | |
|  | | **4 [3,4]** | | | | | | | | | |
|  | | **Strongly Disagree** | | **Disagree** | | **Neither agree nor disagree** | | **Agree** | | **Strongly Agree** | |
| 5. Participating in the intervention fitted well with my existing work. | | 2  3.08% | | 1  1.54% | | 13  20.00% | | 34  52.31% | | 15  23.08% | |
|  | | **4 [4,4]** | | | | | | | | | |
| 6. Delivering this intervention made my job more complicated.* | | 9  13.85% | | 32  49.23% | | 18  27.69% | | 5  7.69% | | 1  1.54% | |
|  | | **2 [2,3]** | | | | | | | | | |
| 7. Participating in the intervention took away time from providing care to patients.* | | 12  18.46% | | 41  63.08% | | 9  13.85% | | 2  3.08% | | 1  1.54% | |
|  | | **2 [2,2]** | | | | | | | | | |
| 8. Delivering this intervention added value to attending to the patient. | | 2  3.08% | | 2  3.08% | | 16  24.62% | | 37  56.92% | | 8  12.31% | |
|  | | **4 [3,4]** | | | | | | | | | |
| 9. I feel there was a need for the HS2 intervention. | | 1  1.56% | | 3  4.69% | | 17  26.56% | | 34  53.13% | | 9  14.06% | |
|  | | **4 [3,4]** | | | | | | | | | |
| 10. The HS2 leaflet met my patients’ needs. | | 3  4.62% | | 5  7.69% | | 16  24.62% | | 33  50.77% | | 8  12.31% | |
|  | | **4 [3,4]** | | | | | | | | | |
| 11. I felt that patients did not find the HS2 intervention helpful.* | | 4  6.15% | | 28  43.08% | | 23  35.38% | | 9  13.85% | | 1  1.54% | |
|  | | **3 [3,2]** | | | | | | | | | |
| 12. The HS2 intervention was not necessary for good patient care.* | | 7  10.94% | | 34  53.13% | | 17  26.56% | | 6  9.38% | | 0  0% | |
|  | | **2 [2,3]** | | | | | | | | | |
| 13. I feel that my organisation’s culture of safe care supported  implementation | | 1  1.67% | | 6  10.00% | | 13  21.67% | | 35  58.33% | | 5  8.33% | |
|  | | **4 [3,4]** | | | | | | | | | |
| 14. I believe my  organisation's improvement culture supported implementation of the intervention. | | 1  1.67% | | 5  8.33% | | 17  28.33% | | 31  51.67% | | 6  10.00% | |
|  | | **4 [3,4]** | | | | | | | | | |
| 15. Implementing HS2 has not affected my organisation's patient safety culture | | 1  1.67% | | 9  15.00% | | 26  43.33% | | 19  31.67% | | 5  8.33% | |
|  | | **3 [3,4]** | | | | | | | | | |
| 16. Implementing HS2 has not affected my organisation's quality improvement | | 1  1.67% | | 11  18.33% | | 28  46.67% | | 16  26.67% | | 4  6.67% | |
|  | | **3 [3,4]** | | | | | | | | | |
| 17. I plan to continue giving out the leaflet. | | 1  1.69% | | 1  1.69% | | 6  10.17% | | 32  54.24% | | 19  32.20% | |
|  | | **4 [4,5]** | | | | | | | | | |
| 18. It will be difficult to  implement this leaflet in other ambulance services.* | | 12  20.34% | | 28  47.46% | | 16  27.12% | | 3  5.08% | | 0  0% | |
|  | | **2 [2,3]** | | | | | | | | | |
|  | **Yes** | | **No** | | **Can’t remember** | |  | |  | |  |
| 19. Did you receive training to help you implement the intervention? | 6  10.17% | | 42  71.19% | | 11  18.64% | |  | |  | |  |
|  | **2 [2,2]** | | | | | |  | |  | |  |
|  | **Strongly Disagree** | | **Disagree** | | **Neither agree nor disagree** | | **Agree** | | **Strongly Agree** | |  |
| 20. I was satisfied with the training I received. | 0  0% | | 0  0% | | 3  50% | | 1  16.67% | | 2  33.33% | |  |
|  | **3.5 [3,5]** | | | | | | | | | |  |
| 21. The training was timely. | 1  16.67% | | 0  0% | | 3  50.00% | | 1  16.67% | | 1  16.67% | |  |
|  | **3 [3,4]** | | | | | | | | | |  |
| 22. The training was not very relevant.* | 3  50.00% | | 1  16.67% | | 2  33.33% | | 0  0% | | 0  0% | |  |
|  | **1.5 [1,3]** | | | | | | | | | |  |
| 23. The training was sufficient. | 0  0% | | 0  0% | | 2  33.33% | | 2  33.33% | | 2  33.33% | |  |
|  | **4 [3,5]** | | | | | | | | | |  |
| 24. I would have liked to have received  more training.* | 0  0% | | 0  0% | | 3  50% | | 3  50% | | 0  0% | |  |
|  | **3.5 [4,5]** | | | | | | | | | |  |
| 25. I needed support when delivering the intervention.* | | 12  20.34% | | 34  57.63% | | 4  6.78% | | 7  11.86% | | 2  3.39% | |
|  | | **2 [2,2]** | | | | | | | | | |
| 26. Colleagues could help  with the intervention if I needed this. | | 1  1.69% | | 3  5.08% | | 12  20.34% | | 36  61.02% | | 7  11.86% | |
|  | | **4 [3,4]** | | | | | | | | | |
| 27. Colleagues could answer questions on  HS2 if I needed this. | | 1  1.69% | | 4  6.78% | | 10  16.95% | | 40  67.80% | | 4  6.78% | |
|  | | **4 [4,3]** | | | | | | | | | |
| 28. I think that this intervention has been  beneficial to patients. | | 1  1.72% | | 4  6.90% | | 2  3.45% | | 41  70.69% | | 10  17.24% | |
|  | | **4 [4,4]** | | | | | | | | | |
| 29. I think that delivering the HS2 leaflet will not prevent recurrent hypoglycaemia episodes.* | | 2  3.45% | | 22  37.93% | | 15  25.86% | | 16  27.59% | | 3  5.17% | |
|  | | **3 [2,4]** | | | | | | | | | |
| 30. I think that  delivering the HS2 leaflet will reduce repeat ambulance  attendances | | 3  5.17% | | 16  27.59% | | 15  25.86% | | 21  36.21% | | 3  5.17% | |
|  | | **3 [2,3]** | | | | | | | | | |
| 31. I think that the HS2 leaflet will not enhance patient self-care.* | | 5  8.62% | | 30  51.72% | | 9  15.52% | | 10  17.24% | | 4  6.90% | |
|  | | **2 [2,3]** | | | | | | | | | |
| 32. Implementing this leaflet has improved my feeling that quality improvement. | | 2  3.51% | | 1  1.75% | | 13  22.81% | | 34  59.65% | | 7  12.28% | |
|  | | **4 [3,4]** | | | | | | | | | |
| 33. I had doubts about taking part in this implementation before it started.* | | 5  8.77% | | 23  40.35% | | 22  38.60% | | 6  10.53% | | 1  1.75% | |
|  | | **3 [2,3]** | | | | | | | | | |

Medians and IQRs are shown in brackets. *Items are reversely scored.

**Table S4 Scoring of each item on a scale from 1 to 5 of the PWD survey.**

|  | **Strongly Disagree** | **Disagree** | **Neither agree nor disagree** | **Agree** | **Strongly Agree** |
| --- | --- | --- | --- | --- | --- |
| 1. It was easy to understand how to use the leaflet. | 1  10% | 0  0% | 1  10% | 4  40% | 4  40% |
|  | **4 [4,5]** | | | | |
| 2.It was difficult to follow the advice given to me by the ambulance staff* | 6  60% | 2  20% | 2  20% | 0  0% | 0  0% |
|  | **5 [4,5]** | | | | |
| 3.I was confident following the advice given to me. | 0  0% | 0  0% | 3  25% | 4  33.3% | 5  41.7% |
|  | **4 [3.5,5]** | | | | |
| 4.The ambulance staff were there to help me answer questions, if I needed to. | 0  0% | 0  0% | 2  16.7% | 5  41.7% | 5  41.7% |
|  | **4 [4,5]** | | | | |
| 5.The leaflet did not add value to the care I received from the ambulance  Services.* | 3  25% | 6  50% | 3  25% | 0  0% | 0  0% |
|  | **4 [3.5,4.5]** | | | | |
| 6.The advice the ambulance staff gave me added value to the care I received. | 0  0% | 0  0% | 4  33.3% | 3  25% | 5  41.7% |
|  | **4 [3,5]** | | | | |
| 7.I feel that being given this leaflet took  away valuable time from my interaction with staff.* | 2  16.7% | 7  58.3% | 3  25% | 0  0% | 0  0% |
|  | **4 [3.5,4]** | | | | |
| 8.I feel there was a need for the leaflet. | 0  0% | 1  8.3% | 5  41.7% | 4  33.3% | 2  16.7% |
|  | **3.5 [3,4]** | | | | |
| 9.The leaflet did not meet my needs.* | 3  25% | 6  50% | 3  25% | 0  0% | 0  0% |
|  | **4 [3.5,4.5]** | | | | |
| 10.I was not happy to receive this  leaflet and the extra advice was given.* | 4  33.3% | 4  33.3% | 4  33.3% | 0  0% | 0  0% |
|  | **4 [3,5]** | | | | |
| 11.I feel that being given this leaflet has made things easier for me. | 0  0% | 1  8.3% | 6  50% | 1  8.3% | 4  33.3% |
|  | **3 [3,5]** | | | | |
| 12.I have found this leaflet beneficial. | 0  0% | 1  8.3% | 5  41.7% | 3  25% | 3  25% |
|  | **3.5 [3,4.5]** | | | | |
| 13.I think that having this leaflet will not prevent another hypo attack.* | 1  9.1% | 3  27.3% | 7  63.7% | 0  0% | 0  0% |
|  | **3 [3,4]** | | | | |
| 14.I think that having this leaflet will increase the possibility that I  will have a hypo. | 3  27.3% | 3  27.3% | 5  45.5% | 0  0% | 0  0% |
|  | **4 [3,5]** | | | | |
| 15.Receiving the leaflet reminded/encouraged me to have a chat/check up with my GP. | 0  0% | 2  18.9% | 7  63.6% | 0  0% | 2  18.9% |
|  | **3 [3,3]** | | | | |
| 16.Receiving the leaflet reminded/encouraged me to have a  chat/check up with  my Diabetes Cons. | 1  9.1% | 2  18.2% | 3  27.3% | 3  27.3% | 2  18.2% |
|  | **3 [2,4]** | | | | |
| 17.Receiving the leaflet reminded/encouraged me to adjust  my medication. | 0  0% | 4  40% | 4  40% | 0  0% | 2  20% |
|  | **3 [2,3]** | | | | |
| 18.Receiving the leaflet reminded/encouraged me to test my blood glucose more often | 1  9.1% | 2  18.2% | 2  18.2% | 4  36.4% | 2  18.2% |
|  | **4 [2,4]** | | | | |
| 19.Receiving the leaflet reminded/encouraged me to adjust my diet. | 0  0% | 4  36.4% | 2  18.2% | 3  27.3% | 2  18.2% |
|  | **3 [2,4]** | | | | |
| 20.Receiving the leaflet reminded/encouraged me to avoid  alcohol for 24 hours. | 0  0% | 3  27.3% | 5  45.5% | 1  9.1% | 2  18.2% |
|  | **3 [2,4]** | | | | |
| 21.Receiving the leaflet reminded/encouraged me to reduce  my overall alcohol consume. | 0  0% | 3  30% | 4  40% | 1  10% | 2  20% |
|  | **3 [2,4]** | | | | |
| 22.Receiving the leaflet reminded/encouraged me to exercise more. | 1  9.1% | 2  18.2% | 7  63.6% | 0  0% | 1  9.1% |
|  | **3 [2,3]** | | | | |
| 23.Receiving the leaflet reminded/encouraged me to not to drive or operate machinery. | 0  0% | 0  0% | 8  80% | 0  0% | 2  20% |
|  | **3 [3,3]** | | | | |
| 24.Receiving the leaflet reminded/encouraged me to avoid  strenuous activity  for 24. | 0  0% | 0  0% | 7  70% | 1  10% | 2  20% |
|  | **3 [3,4]** | | | | |
| 25.I believe that leaflets have the ability to change everyday behaviour of patients. | 0  0% | 1  9.1% | 3  27.3% | 5  45.5% | 2  18.2% |
|  | **4[3,4]** | | | | |
| 26.Having received this leaflet, I no longer  believe that leaflets have the ability to change behaviour.* | 3  33.3% | 4  44.4% | 2  22.2% | 0  0% | 0  0% |
|  | **4[4,5]** | | | | |
| 27.Having received this leaflet, my behaviour  has changed for the better. | 0  0% | 0  0% | 7  70% | 1  10% | 3  20% |
|  | **3[3,4]** | | | | |
| 28.Having received advice from the ambulance staff, has not changed my behaviour.* | 3  33.3% | 4  44.4% | 2  22.2% | 0  0% | 0  0% |
|  | **4[4,5]** | | | | |
| 29.I had doubts about the benefits of the leaflet.* | 3  30% | 4  40% | 3  30% | 0  0% | 0  0% |
|  | 1. **[3,5]** | | | | |

Medians and IQRs are shown in brackets. *Items are reversely scored.
